# Community-based strategies to strengthen men’s engagement in the HIV care cascade in sub-Saharan Africa

**DOI:** 10.1371/journal.pmed.1002262

**Published:** 2017-04-11

**Authors:** Monisha Sharma, Ruanne V. Barnabas, Connie Celum

**Affiliations:** 1 Department of Epidemiology, University of Washington, Seattle, Washington, United States of America; 2 Department of Global Health, University of Washington, Seattle, Washington, United States of America; 3 School of Medicine, University of Washington, Seattle, Washington, United States of America; 4 Vaccine and Infectious Diseases Division, Fred Hutchinson Cancer Research Center, Seattle, Washington, United States of America

## Abstract

Monica Sharma and colleagues discuss evidence-based approaches to improving HIV services for men in sub-Saharan Africa.

**Summary points**

- Men in sub-Saharan Africa are less likely than women to engage in HIV services across the care cascade, resulting in poorer clinical outcomes.
- Health care facilities have achieved limited HIV testing and treatment coverage in men, with barriers including confidentiality concerns, distance to the facility, inconvenient hours, and perceptions that facilities provide women-centered services. Other barriers to male engagement include stigma, poverty, and feelings of compromised masculinity associated with seeking health care.
- Community-based HIV interventions can overcome barriers associated with facilities and increase men’s engagement in care. Social and livelihood interventions can reduce stigma and poverty.
- Community-based testing interventions (particularly home and mobile) have high acceptability and reach more men than health care facility-based approaches. For men testing HIV positive, providing immediate antiretroviral therapy (ART) is associated with high retention and viral suppression. This strategy of “collapsing the cascade” provides streamlined services and reduces loss to follow-up.
- Community-based interventions should be tailored to the needs of men to maximize uptake, including flexible hours, multiple follow-up visits, and convenient and private access to care. Integrating HIV testing into screening for chronic disease can reduce stigma and increase program efficiency. More research is needed on male-centered approaches to increase men’s engagement in HIV services, particularly later in the cascade. Interventions targeted to men who have sex with men are urgently needed.
- The current state of evidence strongly suggests that community-based test-and-treat strategies can reduce the gender disparity in HIV testing and treatment by achieving higher levels of ART coverage and viral suppression in HIV-positive men.

## Introduction

The successful scale-up of antiretroviral therapy (ART) in sub-Saharan Africa (SSA) will require policy makers to address the gender gap in HIV testing and treatment access. Men in SSA are less likely than women to undergo HIV testing and more likely to start ART at advanced disease stages and interrupt or drop out of ART [1]. These disparities have resulted in a life expectancy gap of up to 10 years between HIV-positive men and women [2–5]. Low male testing and treatment rates also increase HIV transmission to female partners. For example, pregnant women in SSA have high HIV testing coverage through antenatal care (ANC) yet have twice the HIV incidence of nonpregnant women [6]. This can be partially attributable to low testing rates in their male partners. In Kenya during 2013, 88% of pregnant women were tested for HIV compared to 4.5% of their male partners [7,8].

Men who have sex with men (MSM) are an underserved group who should not be overlooked. Despite having an HIV prevalence up to eight times higher than the general population, HIV interventions for MSM in SSA are scarce [9,10]. Numerous policy barriers exist that restrict availability and access to HIV-related services for MSM, including police harassment and criminal laws [11].

Stigma is one potential explanation for low male engagement in the HIV care cascade. HIV-positive persons are often perceived by their community as disabled and incapable of contributing economically to society [12]. Men may be more affected by this stigma as they are traditionally the family “breadwinners.” Poverty and food insecurity, common in SSA, can exacerbate HIV-related stigma as engagement in the labor force is crucial for community survival [12]. Poverty can also directly affect engagement in care as individuals lack resources required to attend facilities. Further barriers to visiting clinics include confidentiality concerns, costs (transport, wait time, and lost wages), inconvenient hours, and the perception that clinics are places for women [13].

## Lack of high-quality data on male HIV prevalence and health-seeking behavior can hinder prevention strategies

Accurate HIV prevalence estimates are crucial for designing and evaluating prevention programs. However, HIV surveillance programs such as Demographic and Health Surveys (DHS) are expensive to implement. Instead, much of SSA relies on prevalence estimates extrapolated from ANC data, which exclude men altogether [14]. Countries with DHS tend to have more accurate HIV prevalence estimates. However, measuring male HIV prevalence can be difficult as disproportionately more men decline DHS participation (although nonparticipation is an issue for both sexes). Studies show that DHS often underestimate HIV prevalence, indicating nonparticipation is related to knowledge of HIV status [15–17]. An analysis of 12 DHS found that 10 of the 12 surveys underestimated male HIV prevalence, with bias-corrected estimates up to 9% higher than original DHS prevalence estimates [15]. Another study evaluating DHS in Zambia found that the sex gap in HIV prevalence disappeared after correcting for nonparticipation, with prevalence in men increasing from 12% to 21% [16]. Underestimating HIV prevalence can lead to prevention policies that do not adequately address the male HIV burden. Advanced methods to adjust HIV prevalence for nonparticipation including instrumental variables and Heckman-type selection models can generate more accurate estimates [15,17,18]. These methods will become increasingly important as knowledge of HIV status increases in SSA, which can increase the bias in DHS estimates [14]. Additionally, bias correction is one of the few viable options for obtaining accurate HIV prevalence estimates in settings such as the Swaziland HIV Incidence Measurement Survey (SHIMS), which had lower response rates for men (65%) compared to women (81%) despite up to nine repeated home visits to reduce nonparticipation in the survey [19].

Data on HIV prevalence and health-seeking behaviors (voluntary medical male circumcision [VMMC] and ART uptake and adherence) also inform mathematical models that evaluate HIV interventions. A validation study assessing the ability of 10 mathematical models to predict HIV burden in South Africa found that 8 out of 10 models projected declining male HIV prevalence, whereas empirical data showed increasing prevalence; this may be partly because the models assumed equal ART uptake among men and women [20]. Future models should account for sex differences in HIV testing, ART initiation, and retention to more accurately estimate the impact of interventions.

Ascertaining where losses to follow-up occur across the HIV care cascade is also crucial to targeting interventions. However, it is challenging to generate accurate HIV-related mortality estimates since many HIV-positive men never access care. Additionally, clinics often do not track outcomes for persons who have dropped out of care, making it difficult to determine if men have transferred to another facility or stopped treatment [21]. A study conducted in South Africa was able to assess mortality throughout the HIV continuum using a population-based cohort and determining cause of death in all persons, including those not engaged in HIV care [22]. The authors found that the gap between female and male life expectancy doubled from 2003 to 2011 and twice as many women initiated ART than men. Over half (57%) of male HIV mortality in 2011 occurred among individuals who never sought care, highlighting the importance of HIV testing and linkage strategies. However, substantial proportions of men were lost throughout the cascade, including after ART initiation (33%), so interventions to maintain retention are vital; otherwise, increasing HIV testing and linkage could shift the distribution of deaths to later in the cascade instead of preventing them entirely. Improving ART adherence and retention can also reduce the risk of developing and transmitting drug-resistant virus.

## Community-based HIV testing and counseling (HTC) strategies are successful at reaching men

Facility-based HTC has achieved limited coverage in men. Studies highlight men’s desire to test outside clinics [23], with some men preferring to test at home [24], while others have concerns about the confidentiality of home testing [25] and consider mobile HTC to be more private [26]. As there is no one-size-fits-all approach, a variety of community-based HTC strategies will likely be needed to achieve high testing coverage (Table 1). Meta-analyses find that community HTC achieves higher population coverage than facility testing [27], with home and mobile HTC increasing coverage in men (mobile having a larger effect than home HTC) [26]. Community HTC reaches more first-time testers, suggesting expanded coverage to persons who may not otherwise undergo facility testing [28]. Additionally, community HTC identifies asymptomatic HIV-positive men at higher cluster of differentiation 4 (CD4) cell counts [28]. This can facilitate earlier linkage to ART, preventing morbidity, mortality, and transmission.

**Table 1:** Community-based HIV HTC interventions to increase male testing and engagement in care.

Intervention	Description	Results
Active partner notification services (APSs)	Exposure notification and HTC for sexual partners of newly diagnosed HIV-positive persons (index cases)	Three APS trials in SSA found high testing uptake, HIV positivity, and CD4 count in male partners. In Cameroon, APS was scaled up by an NGO, and 66% of reported partners were tested, of which 50% tested HIV positive (similar results for men and women) [29]. An RCT in Malawi reached 51% of reported partners using provider and contract referral (64% HIV positivity, median CD4 count 344 cells/uL) [30]. An APS community RCT in Kenya achieved 67% coverage in the intervention arm compared to 13% facility testing in the control arm [31].
Mobile HIV testing and counseling	HTC provided in mobile vans or containers located in central areas of a community	A community RCT conducted in SSA (Project Accept) showed a 45% increase in male HIV testing in intervention compared to control communities. The intervention decreased the baseline testing coverage gap between men and women. Social norms regarding HIV testing also moderately improved (6% higher in intervention communities), with a larger effect found in men compared to women (8% versus 4%) [32].
Home HIV testing and counseling	Door-to-door HTC offered in a catchment area	An RCT of home HTC in South Africa tested 38% of men in the target population (although coverage was higher in women: 57%) [33]. The intervention provided clinic referral to immediate and universal ART. Overall, 48% of HIV-positive men linked to care within 6 months, and 63% within 12 months (similar results for women).
Multidisease campaigns	Short health campaigns (usually mobile) providing HTC integrated with health promotion/screening for other diseases	The SEARCH community RCT achieved 86% testing coverage in men, with a high percentage of first-time testers (43%) and high CD4 count at testing (514 cells/uL). The SEARCH intervention also provided immediate and universal linkage to ART, which achieved 80% viral suppression in men in 2 years [34].

NGO, nongovernmental organization; RCT, randomized controlled trial; SEARCH, Sustainable East Africa Research on Community Health.

However, community HTC should be tailored to the needs of men to optimize coverage and effectiveness. For example, a home HTC intervention in Botswana reached 85% of women in the target population compared with 50% of men, likely because the intervention was conducted during the workday, so employed men were missed [35]. Similarly, a home HTC trial (largely conducted during weekdays) successfully contacted more women than men in the target population to offer HTC (74% versus 50%). However, HTC acceptance rates among men contacted were similar to the women who were contacted (77%) [33]. Home HTC with flexible hours and multiple follow-up visits has the potential to test more men. Similarly, a randomized controlled trial (RCT) of workplace HTC for men had 53% uptake when testing was offered on-site compared to 19% for off-site testing, underscoring the importance of convenient testing access [36]. Finally, partner notification (notifying sexual partners of newly diagnosed HIV-positive individuals of their potential exposure and offering HTC) reaches more men when using active (provider tracing or contract referral) compared to passive notification (index cases are asked to notify sexual partners and refer them to the clinic). In a partner notification study in Malawi, men represented only 15% of those presenting to the clinic for testing through passive referral compared to 50% of those tested through active tracing, with no increase in adverse events (e.g., intimate partner violence) [30].

Mobile HTC is a promising strategy for reaching men. A large community RCT in Tanzania and Zimbabwe (Project Accept) reported male testing coverage of 44%–53% in sites randomized to mobile testing compared to 4%–9% facility testing coverage in control sites, decreasing the sex gap in testing coverage found at baseline [37]. Integrating HTC into mobile multidisease campaigns has also had success in reaching men [38]. Community health campaigns can achieve rapid HTC coverage in short time frames (e.g., 2 weeks) [39,40]. The Sustainable East Africa Research on Community Health (SEARCH) campaign conducted in Uganda and Kenya provided HTC; men’s health stations (including prostate cancer screening and VMMC information); screening and treatment for diabetes, hypertension, and malaria; and home HTC for persons who did not participate in the campaign (ClinicalTrials.gov: NCT01864603). This hybrid approach tested an impressive 86% of male community residents, with the vast majority testing through the campaigns (80%) [38]. Combining HTC with other health interventions can reduce HIV-related stigma while improving program efficiency. Studies find that men prefer HIV services that are not separate from other health care services [41].

HTC for male partners of pregnant woman is another strategy to expand male testing. Messaging that testing can protect one’s unborn child can motivate men to undergo HTC [42]. Additionally, couples HTC and status disclosure can increase women’s adherence to ART and regimens for prevention of mother-to-child transmission [43]. Interventions to encourage male partners to come to the ANC for couples HTC have had varied success [44]. Home HTC for male partners of pregnant women can overcome barriers of male ANC attendance and achieve high testing coverage. The Home-Based Partner Education and Testing (HOPE) intervention in Kenya found that 87% of male partners had been tested 6 weeks postpartum in the home-testing arm compared to 39% in the ANC arm [45]. HIV self-testing kits are also potentially effective in reaching men. An RCT in Kenya found that distributing HIV self-tests to pregnant and postpartum women achieved 91% testing coverage in male partners within 3 months compared to 51% in the control arm (male invitation for clinic testing). Self-reported couples testing and disclosure was higher in the self-testing (75%) arm compared to the control arm (35%) [46]. Couples-based approaches may increase male testing regardless of female pregnancy status. For example, a six-session couples behavioral intervention in South Africa found higher couples testing in the intervention compared to the control arm (45% versus 12%) [47].

Community-based educational initiatives to reduce stigma, decrease high-risk sexual behavior, increase HIV testing, and challenge gender norms have had some success (Table 2). The SASA! trial was particularly effective: it was associated with an increase in male HIV testing, condom use, and male appreciation for women’s work [48]. Studies show that social networks are important influences on male HIV testing uptake. Men who believe that at least one close friend has tested for HIV have twice the odds of having undergone HIV testing [49]. More research is needed on social interventions to increase testing coverage in men in SSA. Particularly, training peer leaders to encourage HIV testing should be explored in future studies. Additionally, support from men’s primary female partners has been shown to help overcome barriers to ART initiation and promote adherence [50]. Future interventions should evaluate leveraging social support from primary partners to encourage male engagement in care.

**Table 2:** Community-based educational initiatives for HIV prevention.

Study	Setting / target population	Study design	Intervention	Results
Grassroots soccer [51]	Cape Town, South Africa / unemployed young men (age: 15–25 years)	Cluster RCT	Males were randomized by neighborhood to intervention with access to soccer program, random rapid alcohol and drug testing, and opportunity to enter vocational training program or control (delayed intervention)	Intervention decreased alcohol and drug use by 19% (absolute increase) and increased employment by 20% (absolute increase). No impact on HIV testing, risky sexual behavior, controlling attitudes towards women, or measures of violence.
SASA! [48]	Kampala, Uganda / men and women (average age: 27 years)	Cluster RCT	Community mobilization to reduce IPV and address power imbalances between men and women	Men in the intervention communities were significantly more likely to report HIV testing (aRR 1.50), condom use (aRR 2.03), and fewer concurrent partners (aRR 0.60). They were also more likely to report increased joint decision making (aRR 1.92), greater male participation in household tasks (aRR 1.48), and more open communication and greater appreciation of their partner’s work inside the home (aRR 1.31).
STD risk reduction intervention [52]	Cape Town, South Africa / men aged 18–45 years recruited from taxi stands, bars, and other venues (average age: 27 years)	Cluster RCT	Three-session educational intervention that included interactive exercises, games, role playing, take-home assignments, and educational videos	The odds of consistent condom use were 30% higher in intervention group after 12 months. No difference in odds of multiple sexual partnerships. HIV testing behavior was not assessed.
Stepping Stones [53]	Eastern Cape, South Africa / men and women aged 16–23 years	Cluster RCT	Thirteen-session group facilitated educational intervention (50 hours) held separately for men and women to improve sexual health through more equitable gender relationships	After 2 years, HSV2 incidence was 33% lower in the intervention arm (same reduction for men and women). Men in the intervention were 38% less likely to perpetrate IPV. There was no difference in HIV incidence between arms.
SHARE Project [54]	Rakai, Uganda / men and women aged 15–49 years	Cluster RCT	Violence reduction intervention integrated into routine HIV counseling and testing. Males also received information on alcohol and risky sexual behavior	Women’s self-reported IPV was lower in the intervention compared to control arm (12% versus 16%). Self-disclosure of HIV status was higher in the intervention arm (both sexes). Men in the intervention arm had 41% lower adjusted HIV incidence than the control arm (similar reduction for women).

aRR, adjusted risk ratio; HSV2, herpes simplex virus 2; IPV, intimate partner violence; SHARE, Safe Homes and Respect for Everyone; STD, sexually transmitted disease.

HIV self-testing is a relatively low-cost strategy that achieves high uptake in men, particularly young men [27,55–57]. Self-testing was the preferred option for future HIV testing among men surveyed in Malawi [55]. HIV self-testing can overcome concerns regarding confidentiality of provider-administered HTC and stigma associated with facility testing [55]. However, HIV self-testing is a screening tool and HIV positive results require confirmatory testing. Further research is needed on quality, accuracy, distribution channels, and linkage to care after HIV self-testing.

The literature on HIV testing strategies for MSM in SSA is very limited [10,58,59]. Studies show high HIV positivity combined with a high proportion of first-time testers, highlighting the need for service expansion. Successful testing strategies for MSM are community based (particularly mobile), as many MSM are marginalized without adequate access to conventional health systems [59]. Community HTC for MSM has higher acceptability and greater HIV yield than clinic referral [10]. In addition, HIV self-testing is a potential strategy to reach MSM; it is considered convenient and private [60]. Further, self-testing is useful for groups who benefit from frequent retesting [55].

## Community-based facilitated linkage and retention strategies can increase male engagement in HIV care

The success of HTC strategies depends on their ability to link and engage HIV-positive individuals in care. Extra support may be needed to ensure linkage in community-based modalities, as they are conducted outside of health care systems [27]. Community HTC with facilitated linkage (e.g., counsellor follow-up) has achieved higher linkage than HTC without such support [27]. However, facility linkage rates after community HTC remain lower than what is needed for epidemic control [33,61]. Although community HTC reduces barriers to receiving an HIV test, individuals still must travel and wait at a clinic to obtain treatment. Community-based ART initiation is a convenient alternative to facility linkage. An RCT in Malawi found higher ART uptake with optional home ART initiation compared to facility initiation, with no difference in retention at 6 months [57]. Notably, a recent systematic review found no current or planned trials evaluating community-based ART initiation in men in SSA; likewise, there is a lack of interventions for MSM [62].

Similar to ART initiation, retention is improved with streamlined services. Interviews with persons who have dropped out of care find that lack of transportation is the most common reason for dropout, followed by lack of money and work commitments [21]. Distance from clinic has been negatively associated with ART retention [63]. Food insecurity and long wait times are also reported as barriers to ART pickup [50]. Community-based ART delivery (dispensing ART outside health care systems) may overcome these barriers and increase retention. Home, workplace, or mobile ART pickup sites can be particularly convenient for men. An ongoing RCT, Delivery Optimization for Antiretroviral Therapy (DO ART; ClinicalTrials.gov: NCT02929992), is investigating the effectiveness of community ART initiation and delivery on ART uptake, time to initiation, and viral suppression in South Africa and Uganda.

Similarly, the SEARCH hybrid mobile and home HTC campaign achieved high ART linkage in rural East Africa by “collapsing the cascade” or providing immediate universal treatment for all HIV-positive persons. The campaign offered participants ART visits every 3 months with flexible scheduling, viral load counseling, and treatment for other diseases using a stigma-reducing chronic care model. At baseline, fewer men than women were virally suppressed (44% versus 50%, respectively), but this gap decreased after 2 years of follow-up, with little difference in viral suppression by gender (80% of men and 83% of women) [34].

Since men are more likely than women to be lost to follow-up after testing HIV positive, peer support groups can encourage engagement in care. Studies show men can experience more stigma than women after an HIV diagnosis; they can feel that their masculinity is compromised by admitting they are sick and asking for help [64,65]. Fear of losing respect or being perceived as a failure for acquiring HIV is also a barrier [66]. Support groups and peer counselling can be difficult for men as they may be viewed as activities for women. Peer support groups for men should emphasize responsible fatherhood and skills training. Messaging that ART can allow men to regain their health, restore masculinity compromised by HIV, and provide for their families (particularly children) can be powerful motivators for engagement in care [64,65]. Receiving livelihood support (e.g., farm work, nutritional support, and school fee grants) from providers or support groups can motivate men to initiate and adhere to treatment and also restore masculinity by allowing them to provide for their family [65,66]. Livelihood interventions can also reduce the social stigma caused by an HIV diagnosis [67,68]. Additionally, support groups that challenge gender norms and stigma can change attitudes about masculinity and HIV and increase engagement in care [69]. Effective messages will likely vary by men’s age or the intervention setting. Although ART peer support groups have been successful in motivating engagement and retention, many groups are majority (65%–71%) female [70–72]. Qualitative research on male engagement and retention on ART is sparse.

Financial incentives can increase HIV testing and ART uptake by (1) providing a near-immediate reward for a health behavior and (2) offsetting costs associated with clinic attendance (travel and missed work) [73]. They can also reduce structural HIV risk factors by alleviating poverty and increasing access to education. A study in South Africa investigating incentivized compared to nonincentivized mobile testing for reaching men found higher HIV prevalence (16% versus 5.5%), more first-time testers (60% versus 42%), and more advanced HIV disease (15% versus 8%) in men accessing incentivized compared to nonincentivized services [74]. Fewer studies have examined the effects of cash transfers later in the HIV care cascade, although two trials are ongoing: Link4Health—a combination approach of accelerated ART initiation, counselling, and financial incentives for linkage and retention in Swaziland (ClinicalTrials.gov: NCT01904994)—and ENGAGE4HEALTH—evaluating accelerated ART initiation, short message service (SMS) reminders, and noncash financial incentives for retention and linkage for both sexes in Mozambique (ClinicalTrials.gov: NCT01930084) [73]. More research is needed on conditional and unconditional cash transfers and lottery systems for increasing male engagement in the care cascade. Additionally, some beneficial health behaviors are known to diminish after the cessation of cash transfers, so studies are needed to evaluate the long-term effects of financial incentives on ART adherence [73].

Linkage to care is challenging for MSM since health care workers in SSA typically have little or no training in addressing their health care needs [75]. Further, MSM report discrimination, harassment, and denial of services [76]. A study from Kenya found that providing health care workers with a web-based 2-day MSM sensitivity training decreased homophobic attitudes and increased knowledge of MSM-related health issues (maintained 3 months postintervention). Encouragingly, the strongest impact was seen in those with the most negative attitudes towards MSM [77]. However, such training is rare, and the health care workers who attended the training reported experiencing secondary stigma from other health care workers who were not trained [75]. Therefore, widespread structural interventions are needed to make a substantial impact on health care workers’ attitudes towards MSM. Further, little is known about ART adherence and clinical outcomes in HIV-positive MSM [75]. One of the few studies evaluating this topic found that ART adherence in Kenya was substantially lower in MSM compared to heterosexual men [78]. However, only one intervention to promote engagement in care and adherence to ART in HIV-positive MSM is currently being conducted in SSA [79]. Successful strategies will require multiple approaches including pre-exposure prophylaxis (PrEP), treatment as prevention, and behavioral risk reduction [80].

## Conclusions

More work remains to be done to reduce the gender disparity in HIV testing, linkage, and retention in care in SSA. Multicomponent interventions are needed to reduce stigma and address issues of masculinity and health-seeking behavior. Additionally, leveraging social support and providing poverty alleviation can change norms around testing and treatment while addressing other factors contributing to low engagement in care (e.g., food insecurity and poverty). Community-based testing, and potentially community-based ART initiation and medication resupply, can overcome barriers associated with clinics and strengthen male engagement across the care cascade. A variety of community HTC modalities can be implemented simultaneously to achieve maximum coverage. Targeted messaging to motivate men (e.g., protection of one’s sexual partner and future children, and restoration of health through ART) can increase testing uptake and linkage [81]. Community-based “test and treat” strategies may reduce loss to follow-up associated with clinic-based ART initiation. Further, an integrated approach combining testing and treatment with other HIV interventions (VMMC and PrEP) and chronic disease screening can increase intervention program efficiency while reducing stigma. Further research is needed on community-based interventions that motivate male engagement in care, particularly later in the cascade (i.e., ART initiation and retention).

## Abbreviations

ANC: antenatal care
APS: active partner notification service
ART: antiretroviral therapy
CD4: cluster of differentiation 4
DHS: Demographic and Health Survey(s)
DO ART: Delivery Optimization for Antiretroviral Therapy
HOPE: Home-Based Partner Education and Testing
HTC: HIV testing and counseling
MSM: men who have sex with men
PrEP: pre-exposure prophylaxis
RCT: randomized controlled tria
SEARCH: Sustainable East Africa Research on Community Health
SHIMS: Swaziland HIV Incidence Measurement Survey
SMS: short message service
SSA: sub-Saharan Africa
VMMC: voluntary medical male circumcision
